# The Role of Resting-State Network Functional Connectivity in Cognitive Aging

**DOI:** 10.3389/fnagi.2020.00177

**Published:** 2020-06-12

**Authors:** Hanna K. Hausman, Andrew O’Shea, Jessica N. Kraft, Emanuel M. Boutzoukas, Nicole D. Evangelista, Emily J. Van Etten, Pradyumna K. Bharadwaj, Samantha G. Smith, Eric Porges, Georg A. Hishaw, Samuel Wu, Steven DeKosky, Gene E. Alexander, Michael Marsiske, Ronald Cohen, Adam J. Woods

**Affiliations:** ^1^Department of Clinical and Health Psychology, Center for Cognitive Aging and Memory, College of Public Health and Health Professions, McKnight Brain Institute, University of Florida, Gainesville, FL, United States; ^2^Department of Psychology, McKnight Brain Institute, University of Arizona, Tucson, AZ, United States; ^3^Department of Psychiatry and Neurology, College of Medicine, University of Arizona, Tucson, AZ, United States; ^4^Department of Biostatistics, College of Public Health and Health Professions, College of Medicine, University of Florida, Gainesville, FL, United States; ^5^Department of Neurology, College of Medicine, Center for Cognitive Aging and Memory, McKnight Brain Institute, University of Florida, Gainesville, FL, United States; ^6^Department of Psychiatry, Neuroscience and Physiological Sciences Graduate Interdisciplinary Programs, and BIO5 Institute, University of Arizona and Arizona Alzheimer’s Disease Consortium, Tucson, AZ, United States

**Keywords:** cingulo-opercular, cognitive aging, functional connectivity, resting-state, networks

## Abstract

Aging is associated with disruptions in the resting-state functional architecture of the brain. Previous studies have primarily focused on age-related declines in the default mode network (DMN) and its implications in Alzheimer’s disease. However, due to mixed findings, it is unclear if changes in resting-state network functional connectivity are linked to cognitive decline in healthy older adults. In the present study, we evaluated the influence of intra-network coherence for four higher-order cognitive resting-state networks on a sensitive measure of cognitive aging (i.e., NIH Toolbox Fluid Cognition Battery) in 154 healthy older adults with a mean age of 71 and education ranging between 12 years and 21 years (mean = 16). Only coherence within the cingulo-opercular network (CON) was significantly related to Fluid Cognition Composite scores, explaining more variance in scores than age and education. Furthermore, we mapped CON connectivity onto fluid cognitive subdomains that typically decline in advanced age. Greater CON connectivity was associated with better performance on episodic memory, attention, and executive function tasks. Overall, the present study provides evidence to propose CON coherence as a potential novel neural marker for nonpathological cognitive aging.

## Introduction

By the year 2050, the number of adults over the age of 65 in the United States population is expected to double, signifying one of the fastest-growing age cohorts. With advanced age, crystallized cognitive abilities, such as accumulated knowledge and vocabulary, are maintained and can even improve over time. Conversely, even in the absence of pathology, older adults experience declines in fluid cognitive abilities such as thinking abstractly, reasoning, and decision-making (Salthouse, 2010; Murman, 2015). Declines in these capacities are linked to underlying age-related deficits in processing speed, attention, memory, and executive function (i.e., cognitive aging; Salthouse, 1994, 2000, 2010; Glisky, 2007). Cognitive aging in later life is associated with functional impairments in managing new information, manipulating the environment, and solving problems (Salthouse, 1994, 2000, 2010; Buckner, 2004; Wecker et al., 2005; Harada et al., 2013). These impairments can affect older adults’ ability to function independently within society and the home. Also, extensive evidence reveals that cognitive decline and associated functional decline in older adults has been related to changes in the structure and function of the brain (Salat et al., 1999; Raz, 2000; Reuter-Lorenz et al., 2000; Cabeza, 2002; Raz et al., 2005; Andrews-Hanna et al., 2007; Kennedy and Raz, 2009). Therefore, it is imperative to identify what brain-based factors significantly contribute to cognitive aging. A more comprehensive understanding of age-related neural alterations within cognitive aging may facilitate better differentiation of nonpathological aging from disease states and the identification of target brain areas for intervention.

One of the increasingly popular methods used to study age and pathology-related changes in the brain is resting-state functional magnetic resonance imaging (rs-fMRI). rs-fMRI is used to identify coherent fluctuations in brain activity when a person is not actively engaging in a cognitive task (i.e., at rest; Biswal et al., 2010; Biswal, 2012). rs-fMRI is particularly advantageous when studying aging populations because it allows for the examination of functional connectivity while removing the demand of a task that may be confounded by potential cognitive or motor impairments. Additionally, scan times are relatively short (5–15 min), benefitting older adults that may have difficulties lying on one’s back for long periods or those with psychological concerns regarding a novel scanning environment.

Across the lifespan, rs-fMRI has identified low-frequency resting-state networks that depict the functional architecture of the brain. Resting-state networks have been characterized for aspects of attention (Fox et al., 2006), memory (Vincent et al., 2006), cognitive control (Dosenbach et al., 2007; Vincent et al., 2008; Cole et al., 2010), default mode (Raichle et al., 2001; Buckner et al., 2008), motor (Biswal et al., 1995), and sensory systems (De Luca et al., 2005; Damoiseaux et al., 2006). Yeo et al. (2011) published a parcellation of the brain into seven major resting-state networks: the default mode network (DMN), the dorsal attention network (DAN), the frontoparietal control network (FPCN), the cingulo-opercular network (CON) [commonly referred to as the salience (Seeley et al., 2007) or ventral attention network (Fox et al., 2006)], the limbic network, the visual network, and the somatomotor network.

Investigators have used rs-fMRI techniques to examine how patterns of resting-state network functional connectivity change with age (Andrews-Hanna et al., 2007; Chan et al., 2014; Damoiseaux, 2017; Siman-Tov et al., 2017; Spreng and Turner, 2019). While previous studies have primarily focused on age-related declines in the connectivity of the DMN (Andrews-Hanna et al., 2007; Damoiseaux et al., 2007; Ferreira and Busatto, 2013), recent research has uncovered several additional “higher-order cognitive” networks vulnerable to the aging process (e.g., FPCN, CON, DAN; Geerligs et al., 2015a; Siman-Tov et al., 2017). While it is clear that resting-state network connectivity is disrupted in healthy older adults, it is unclear whether these age-related changes in resting-state functional connectivity contribute to the cognitive aging process. Studies examining the relationship between network connectivity and cognition in older adults have used a variety of cognitive tasks and resting-state methods, producing inconsistent results (see Ferreira and Busatto, 2013 for review). For example, some studies have shown DMN, FPCN, and CON connectivity in older adults to be associated with performance on specific executive functioning, memory, and processing speed tasks (Andrews-Hanna et al., 2007; Damoiseaux et al., 2007; Shaw et al., 2015). However, Onoda et al. (2012) only found an association between CON connectivity and executive functioning (i.e., the Frontal Assessment Battery and Kohs’ Block Design) and did not replicate a relationship between the other higher-order cognitive networks and cognitive performance in older adults. Lastly, Geerligs et al. (2015a) did not find a significant relationship between any of the seven major resting-state networks and cognitive performance in older adults. These inconsistent findings are surprising given the emphasis in the literature on DMN connectivity in aging (Ferreira and Busatto, 2013), suggesting future research should include other networks in analyses and incorporate sensitive cognitive measures that model the cognitive aging process. Importantly, a majority of previous studies compared resting-state connectivity patterns and cognitive performance between younger and older adults. This type of experimental design can be problematic for characterizing connectivity, as it is confounded by cohort effects and age differences in head motion, heart rate variability, and cerebrovascular function (D’Esposito et al., 1999; Geerligs et al., 2015b, 2017; Prins and Scheltens, 2015). Additionally, a majority of these studies had small sample sizes, potentially contributing to null findings. Consequently, there is a need for a study with a large sample of older adults to evaluate the association between sensitive measures of cognitive aging and inter-individual differences in resting-state network functional connectivity.

To comprehensively assess cognitive aging, we used the NIH Toolbox Cognition Battery (Weintraub et al., 2013)^1^. The NIH Toolbox yields a Fluid Cognition Composite, which consists of measures categorized as skills that decline as a function of advanced age: the Dimensional Change Card Sort (executive function), Flanker (executive function and attention), Picture Sequence Memory (episodic memory), List Sorting (working memory), and Pattern Comparison tasks (processing speed). The primary aim was to identify a relationship between resting-state network connectivity and the overall cognitive aging process. As such, this study analyzed the contribution of the four higher-order cognitive networks (DMN, DAN, FPCN, CON) on Fluid Cognition Composite scores in a large sample of healthy older adults. Although the prior literature has been highly variable regarding which networks relate to cognition in older adults, the DMN, FPCN, and CON networks are the most common networks implicated (Andrews-Hanna et al., 2007; Onoda et al., 2012; Shaw et al., 2015) with little evidence for the DAN, which may be less sensitive to the effects of age compared to the other higher-order cognitive networks (Chan et al., 2014; Grady et al., 2016). Therefore, we predicted that older adults with greater within-network resting-state functional connectivity of the DMN, FPCN, and CON networks would have higher Fluid Cognition Composite scores. In secondary analyses, we mapped networks significantly related to the Fluid Cognition Composite onto the subtests to identify which specific domains characterized the relationship between network connectivity and cognitive aging. Our results provide important new insights into age-related cognitive declines and inter-individual variability in resting-state network connectivity, identifying a potential neural marker for nonpathological cognitive aging.

## Materials and Methods

### Participants

Data were collected at baseline from participants recruited for the Stimulated Brain (K01AG050707) and the Augmenting Cognitive Training in Older Adults (ACT, R01AG054077) studies (Woods et al., 2018). Our sample included 159 healthy older adults ranging from 65 to 87 years old (mean age = 71.4 ± 5.1; 92 females; mean education = 16.2 ± 2.4, education range = 12–21 years; Table 1) recruited at the University of Florida (*n* = 110) and the University of Arizona (*n* = 49). Woods et al. (2018) detail the inclusion and exclusion criteria. In brief, participants were between the ages of 65–89, had no history of major psychiatric illness, no history of brain or head injury resulting in loss of consciousness greater than 20 min, and no formal diagnosis or evidence of mild cognitive impairment (MCI), dementia, or neurological brain disease. The Unified Data Set (UDS) of the National Alzheimer’s Coordinating Center (NACC) was used to screen for individuals with possible MCI or dementia (Weintraub et al., 2009). Possible MCI was defined by 1.5 standard deviations below the mean in any of the following domains: general cognition, memory, visuospatial, executive functioning/working memory, or language. All participants were right-handed and had no contraindications for MRI scanning. Before beginning all study procedures, participants signed a consent form approved by the Institutional Review Boards at the University of Florida and the University of Arizona. At the baseline visit, participants completed a variety of cognitive assessments, medical history and mood questionnaires, and an MRI scan. In this study, we used the NIH Toolbox Cognition battery and the rs-fMRI data for our analyses. Three participants were excluded due to incomplete or extreme scores (greater than three standard deviations from the mean) on the NIH toolbox. Additionally, two participants were excluded as outliers due to extreme network connectivity values resulting in a total sample size of 154 older adults.

**Table 1:** Participant demographics and NIH toolbox scores.

Demographics	Mean/SD
Age	71.4 ± 5.1
Education (Number of years)	16.2 ± 2.4
∣rule
**Gender**	**N**
Males	65
Females	94
∣rule
**NIH toolbox**	**Mean/SD**
∣rule
Fluid cognition composite	93.1 ± 8.6
List sorting	98.2 ± 8.9
Pattern comparison	90.6 ± 14.0
Picture sequence memory	95.7 ± 10.2
Flanker	93.9 ± 6.7
Dimensional change card sort	100.9 ± 7.3

*Notes: NIH toolbox scores are unadjusted standard scores*.

### NIH Toolbox

The NIH Toolbox Cognition Battery is a brief set of sensitive measures used to assess a range of cognitive domains (Weintraub et al., 2013). In the present study, we used the unadjusted standard scores for the Fluid Cognition Composite and its five subtests that measure cognitive abilities shown to decline with advanced age. These subtests measure components of executive function, attention, episodic memory, working memory, and processing speed. For instance, the Dimensional Change Card Sort task assesses the set-shifting component of executive function (i.e., the ability to switch among multiple task strategies and rules). Here, a participant must match a target stimulus to a choice stimulus according to the shifting criterion of either shape or color. The Flanker task is a visuospatial attention task that also requires inhibitory control over automatic responses. The goal of this task is to determine the direction of a central target arrow that is flanked by similar stimuli on the left and right. The Picture Sequence Memory task targets episodic memory, a cognitive process involved in the retrieval of learned information. In this task, thematically related pictures are displayed in a sequence, and participants must remember and move the pictures into the sequence demonstrated. The List Sorting task is a measure of working memory, the ability to temporarily hold and manipulate a limited capacity of information. This requires participants to sequence and sort a list of visual and auditory stimuli from smallest to largest increasing in the number of categories and items. Lastly, the Pattern Comparison task is a measure of processing speed, where participants quickly identify whether or not two visual patterns are the same.

### Imaging Acquisition

rs-fMRI data were collected using a 3-Tesla Siemens Magnetom Prisma scanner with a 64-channel head coil at the Center for Cognitive Aging and Memory at the University of Florida and using a 3-Tesla Siemens Magnetom Skyra scanner with a 32-channel head coil at the University of Arizona. Scanner type was included as a covariate in our statistical analyses to control for potential differences in the quality and acquisition of MRI data. Both study sites followed the same scanning procedures and used identical sequences. Participant head motion was constrained by foam padding, and participants were provided with earplugs to reduce the adverse effects of scanner noise. For acquiring resting-state data, participants were asked to rest for about 6 min while keeping their eyes open, as a blood-oxygen-level-dependent (BOLD) scan was acquired with an echo-planar functional protocol [number of volumes = 120, repetition time (TR) = 3,000 ms, echo time (TE) = 30 ms; flip angle = 70°, 3.0 × 3.0 × 3.0 mm^3^ voxels; 44 slices, field of view (FOV) = 240 × 240 mm]. To assist the normalization of the resting-state functional images in the preprocessing stage, high-resolution T1-weighted 3D magnetization prepared rapid acquisition gradient echo (MPRAGE) images were collected (TR = 1,800 ms; TE = 2.26 ms; 1.0 × 1.0 × 1.0 mm^3^ voxels; 176 slices; FOV = 256 × 256 mm; FA = 8°; time = 3 min and 3 s).

### rs-fMRI Preprocessing

Structural and functional images were preprocessed and analyzed using the MATLAB R2016b based functional connectivity toolbox “Conn toolbox” version 18b and SPM 12 (Penny et al., 2007; Whitfield-Gabrieli and Nieto-Castanon, 2012). We followed a preprocessing pipeline which included the functional realignment and unwarping, functional centering of the image to (0, 0, 0) coordinates, slice-timing correction, structural centering to (0, 0, 0) coordinates, structural segmentation and normalization to MNI space, functional normalization to MNI space, and spatial smoothing with a smoothing kernel of 8 mm FWHM. During preprocessing, the Conn toolbox implements an anatomical, component-based, noise correction strategy (*aCompCor*) for spatial and temporal processing to remove physiological noise factors from the data (Behzadi et al., 2007). The implementation of *aCompCor* combined with the quantification of participant motion and the identification of outlier scans through the Artifact Rejection Toolbox (ART)^2^ allows for better interpretation of functional connectivity results (Behzadi et al., 2007; Whitfield-Gabrieli and Nieto-Castanon, 2012; Shirer et al., 2015). The ART was set to the 97th percentile setting with the mean global-signal deviation threshold set at *z* = ±5 and the participant-motion threshold set at 0.9 mm. Due to potential confounding effects, the resulting motion information and frame-wise outliers were included as covariates in our first-level analyses (Behzadi et al., 2007; Power et al., 2012; van Dijk et al., 2012). Applying linear regression and using a band-pass filter of 0.008–0.09 Hz, data were denoised to exclude signal frequencies outside of the range of expected BOLD signals (such as low-frequency scanner drift), minimize participant motion, extract white matter and cerebral spinal fluid noise components, and control for within-participant realignment and scrubbing covariates.

### Within-Network Connectivity and Cognitive Performance Analyses

**Figure 1:**
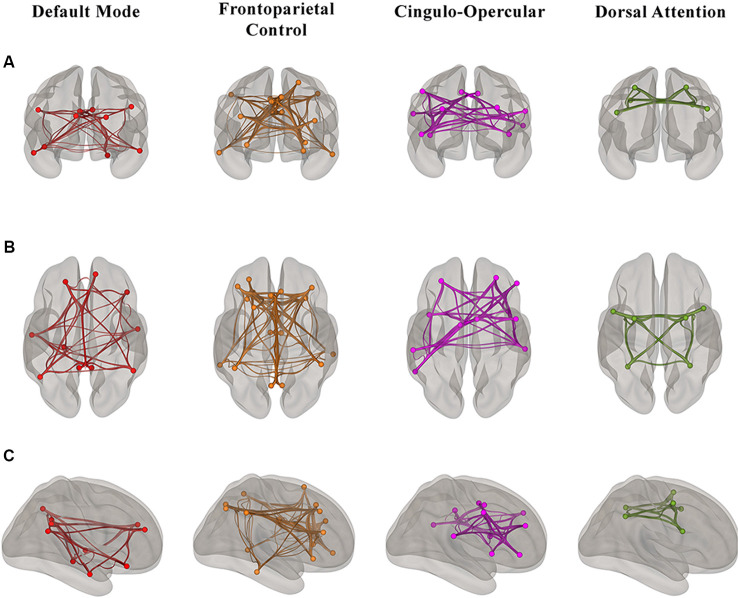
Visualization of the regions of interest (ROI)-ROI connections in each of four higher-order cognitive networks (Yeo et al., 2011) in our sample of healthy older adults from **(A)** anterior **(B)** superior and **(C)** right hemisphere views. Each network is color-coded; however, the colors do not depict different levels of correlation strength.

For the rs-fMRI analyses, we used a publicly available network parcellation of the brain defined by Yeo et al. (2011) that has been commonly used in the resting-state literature (Betzel et al., 2014; Fjell et al., 2017; Khasawinah et al., 2017; Dixon et al., 2018; Dubois et al., 2018; Ruiz-Rizzo et al., 2019). The resting-state networks were projected into MNI152 space, and we specifically defined four of the networks (DMN, DAN, FPCN, and CON) as regions of interests (ROIs) for ROI-ROI functional connectivity analyses. ROI-ROI analyses are Fisher z-transformed bivariate correlations between brain regions’ BOLD time-series that quantify associations in the activation at rest and serve as a proxy for connectivity. Using the CONN toolbox, every participant’s average within-network connectivity was calculated by computing the mean of the pairwise correlations between the specified (Yeo et al., 2011) ROIs that comprised each of the four higher-order cognitive networks (Figure 1).

First, to assess how the four networks contribute to the general domain of cognitive aging, we ran a multiple linear regression evaluating the unique effect of within-network connectivity on Fluid Cognition Composite scores. For secondary analyses, linear regressions were conducted to characterize the resting-state networks’ domain-specific influence in cognitive aging by regressing within-network connectivity onto the cognitive subtests: Dimensional Change Card Sort, Flanker, Picture Sequence Memory, List Sorting, and Pattern Comparison. In the secondary analyses, we only evaluated networks that significantly contributed to Fluid Cognition Composite scores, since our primary question concerns identifying important resting-state networks in the cognitive aging process overall. We controlled for age, education, sex, and scanner type in all of our models. All statistical analyses were performed using SPSS version 25.

## Results

### Primary Analyses: Within-Network Connectivity and Composite Scores

**Figure 2:**
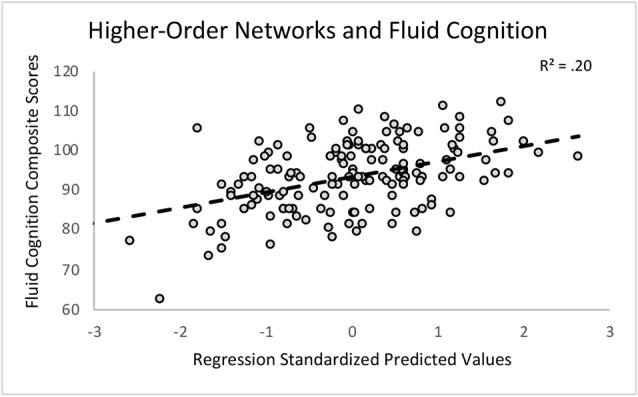
A scatterplot depicting the primary regression analysis with the standardized predicted values (X-axis) resulting from regressing age, sex, education, scanner, and within-network connectivity values of the cingulo-opercular network (CON), frontoparietal control network (FPCN), default mode network (DMN), and dorsal attention network (DAN) on the Fluid Cognition Composite unadjusted standard scores (Y-axis).

**Figure 3:**
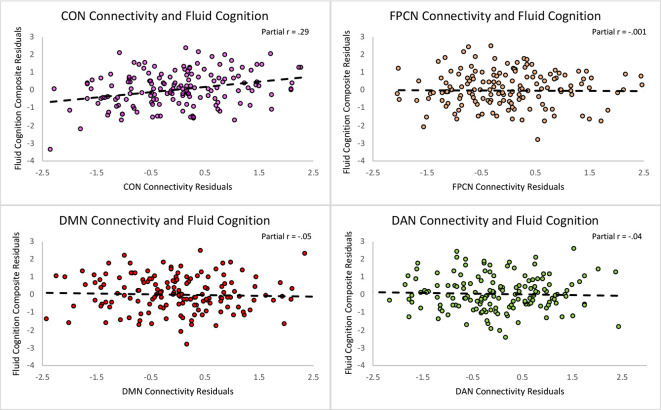
Scatterplots depicting the unique relationships between each network and the Fluid Cognition Composite controlling for the rest of the predictors in the regression. The X and Y axes represent the standardized residuals for the independent and dependent variables, partialling out the effects of the remaining predictors. As such, the slopes reflect partial correlations.

First, we regressed within-network connectivity values for DMN, DAN, FPCN, and CON simultaneously on Fluid Cognition Composite scores to evaluate which network has the greatest influence in the general cognitive aging process. In these primary analyses, age and education were significantly associated with Fluid Cognition Composite scores (*p*-values < 0.01), such that older age was associated with lower composite scores and more years of education was associated with higher composite scores. Conversely, sex and scanner type were not significantly associated with Fluid Cognition Composite scores. Out of the four high-order cognitive networks, only CON within-network connectivity had a significant relationship with Fluid Cognition Composite scores, such that greater CON connectivity was associated with better performance (*R*^2^ = 0.20, *β* = 0.33, *p* < 0.001). The overall model explained 20% of the variance in Fluid Cognition Composite scores, and notably, CON connectivity explained 7.4% of the variance, while age and education explained 3.8% and 5.7%, respectively (Table 2; Figures 2, 3).

**Table 2:** Within-network connectivity and fluid composite score results.

Predictors	*β*	*t*	*p*	*Model R^2^*
Age	−0.21	−2.64	0.01*	0.20
Education	0.25	3.22	0.002*	
Sex	0.05	0.70	0.49	
Scanner	−0.001	−0.02	0.98	
CON	0.33	3.66	<0.001*	
FPCN	−0.01	−0.06	0.96	
DMN	−0.06	0.78	0.61	
DAN	−0.03	0.36	0.78	

Notes: CON, cingulo-opercular network; FPCN, frontoparietal control network; DMN, default mode network; DAN, dorsal attention network. *Significant result p < 0.01.

### Within-Network Connectivity and NIH Toolbox Subtests

**Figure 4:**
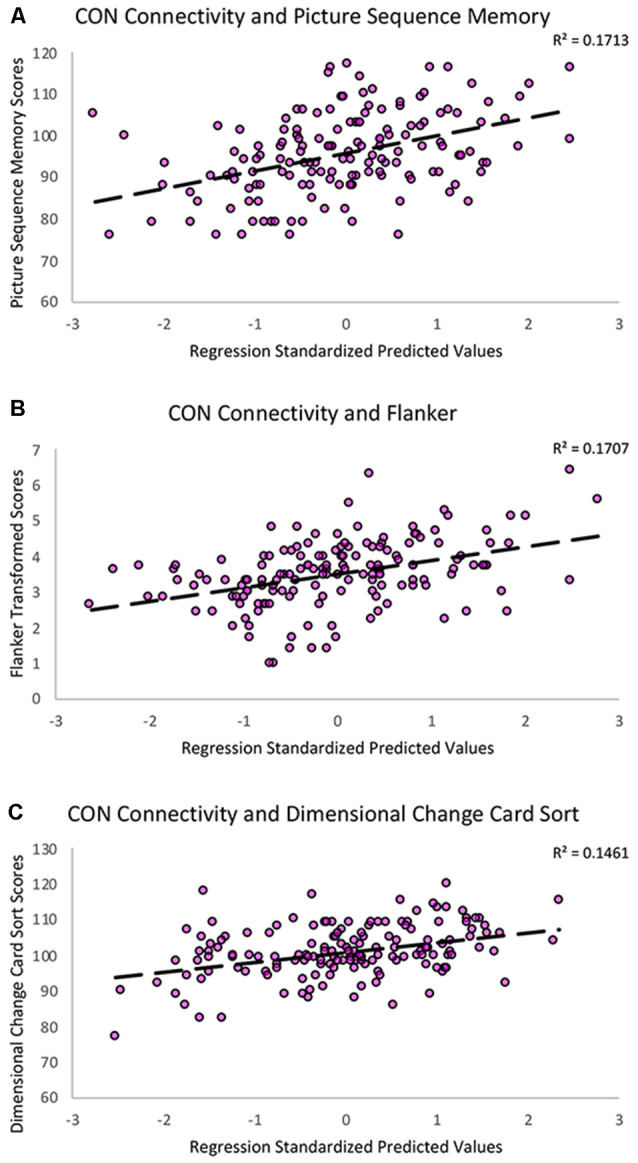
Scatterplots showing the significant relationships between **(A)** Picture Sequence Memory **(B)** Flanker and **(C)** Dimensional Change Card Sort subtest scores and CON within-network resting-state connectivity controlling for age, education, sex, and scanner covariates. The Y-axes reflect the unadjusted standard scores for Picture Sequence Memory and Dimensional Change Card Sort and the square root transformed scores for the Flanker task.

To better characterize the relationship between CON connectivity and cognitive aging, we ran secondary analyses to identify which specific fluid cognitive subtests were associated with the CON network. The distribution of scores on the Flanker subtest was positively skewed; therefore, we performed a square root transformation to meet normality assumptions before analyses. Notably, CON within-network connectivity was related to better performance across three of the five fluid cognition subtests, suggesting the network has a relatively broad relationship with cognitive aging rather than a relationship-driven by a specific domain. Greater CON within-network connectivity was associated with higher scores on Dimensional Change Card Sort (*R*^2^ = 0.15, *β* = 0.26, *p* = 0.001), Flanker (*R*^2^ = 0.17, *β* = 0.29, *p* < 0.001), and Picture Sequence Memory tasks (*R*^2^ = 0.17, *β* = 0.25, *p* = 0.001). CON connectivity was not significantly related to performance on List Sorting (*R*^2^ = 0.07, *β* = 0.10, *p* = 0.20) or Pattern Comparison (*R*^2^ = 0.09, *β* = 0.12, *p* = 0.13; Table 3; Figure 4).

**Table 3:** CON within-network connectivity and NIH toolbox subtest results.

Fluid tasks	*Model R^2^*	*β*	*t*	*p*	*sr^2^*
Flanker	0.17	0.29	3.74	<0.001*	0.08
Picture sequence	0.17	0.25	3.27	0.001*	0.06
DCCS	0.15	0.26	3.33	0.001*	0.06
List sorting	0.07	0.10	1.28	0.20	0.01
Pattern comparison	0.09	0.12	1.54	0.13	0.01

Notes: CON, cingulo-opercular network; DCCS, Dimensional Change Card Sort. *Significant result *p* < 0.01.

In these subtest analyses, age was significantly related to performance on the Pattern Comparison task, such that older age was associated with worse performance (*β* = −0.22, *p* = 0.008). Sex was significantly related to performance on Picture Sequence Memory, such that females performed better than males (*β* = 0.28, *p* < 0.001). Lastly, those with higher education performed significantly better on the List Sorting (*β* = 0.21, *p* = 0.01), Pattern Comparison (*β* = 0.15, *p* = 0.05), Flanker (*β* = 0.23, *p* = 0.004), and Dimensional Change Card Sort (*β* = 0.26, *p* = 0.001) tasks.

## Discussion

Aging is associated with disruptions in the functional architecture of the brain. However, due to previously mixed findings, it is unclear if age-related changes in resting-state network functional connectivity are linked to the cognitive aging process. The present study offers important new insights by uncovering a specific relationship between resting-state network functional connectivity and cognitive performance in a large sample of healthy older adults. Here, we identified a resting-state network involved in general fluid cognition. Additionally, we outlined the cognitive scope of this network by mapping connectivity onto processing speed, episodic memory, working memory, attention, and executive function subdomains. By linking resting-state network connectivity to various aspects of the cognitive aging process, we hope to create a foundation for future targeted intervention strategies. While the literature has focused on the DMN and its implications in Alzheimer’s disease (Buckner et al., 2008; Ferreira and Busatto, 2013; Sullivan et al., 2019), our findings suggest further examination of the CON in the context of nonpathological cognitive aging (Onoda et al., 2012; Geerligs et al., 2015a; Siman-Tov et al., 2017).

### Cognitive Control in Cognitive Aging: Cingulo-Opercular Network

The CON is commonly referred to as one of the cognitive control networks (Cole and Schneider, 2007; Dosenbach et al., 2008). Cognitive control is necessary for flexibly allocating mental resources to produce goal-directed behavior. Examples of control processes include attending to stimuli, preparing and initiating a response, and adapting to feedback (Cole and Schneider, 2007). These components of cognition are necessary for the successful completion of a variety of tasks in daily life. In the present study, CON was the only network that was significantly associated with the NIH Toolbox Fluid Cognition Composite. Beyond the matter of significance, CON intra-network coherence explained more of the variance in composite scores than both age and education. These results suggest that CON connectivity is an important factor that influences fluid cognition and may be a compelling target for novel interventions (e.g., transcranial direct current stimulation) to enhance overall cognitive function in older adults. Additionally, CON within-network connectivity was positively associated with performance on three out of the five subtest domains typically vulnerable to the aging process: episodic memory, attention, and executive function. This pattern of findings suggests the CON network has a relatively global relationship with the cognitive aging process rather than a relationship driven by a specific domain. These results support the notion of the general widespread involvement of the CON network in cognitive control (Dosenbach et al., 2007; Sestieri et al., 2014). Broadly, CON is involved in the implementation and maintenance of the perceptual and attentional information in a task (Dosenbach et al., 2006, 2007, 2008). This is evident through CON’s sustained activation during trial initiation, target detection, task maintenance, and response (Dosenbach et al., 2007, 2008; Sestieri et al., 2014; Han et al., 2018). CON’s expansive functional responsibility is necessary for the brain’s moment-to-moment information processing, a general proficiency greatly affected by age (Salthouse, 2000, 2010). Therefore, our findings suggest that greater functional connectivity within CON at rest may reflect a better ability to properly activate this important network during the execution of fluid cognitive tasks in older adults.

Additionally, CON consists of brain regions important for fluid cognitive abilities like decision-making, planning, target and error detection, updating, and switching (i.e., frontal operculum, medial superior frontal cortex, dorsal anterior cingulate cortex, and anterior insula; Gehring and Knight, 2000; Jung and Haier, 2007; Han et al., 2018). These regions are susceptible to gray matter atrophy and white matter disruptions with age, which contribute to functional activation alterations and declines in cognitive performance (Salat et al., 1999; Raz, 2000; Reuter-Lorenz et al., 2000; Cabeza, 2002; Raz et al., 2005; Andrews-Hanna et al., 2007; Kennedy and Raz, 2009). He et al. (2014) showed that gray matter volume of the insular cortices and the dorsal anterior cingulate, major hubs in the CON network, as well as the functional connectivity of the left insula cortex were associated with scores on the Mini-Mental Status Examination in their sample of older adults. The resting-state functional connectivity of the left insula specifically has also been shown to mediate the association between age and visual processing speed in healthy older adults (Ruiz-Rizzo et al., 2019). Greater CON resting-state connectivity may signify greater maintenance of structural integrity in these regions involved in fluid cognition. Future research should utilize multimodal imaging to further assess the relationship between age-related structural changes and resting-state network functional connectivity.

### Higher-Order Cognitive Networks in Cognitive Aging: Default Mode, Dorsal Attention, Frontoparietal Control, Cingulo-Opercular

CON is a part of a larger group of networks referred to as the higher-order cognitive networks (i.e., CON, FPCN, DMN, DAN). Functional connectivity within these networks typically decreases with age along the same trajectory of age-related structural deterioration and cognitive decline (Park and Reuter-Lorenz, 2009; Giorgio et al., 2010; Geerligs et al., 2015a; Siman-Tov et al., 2017). Conversely, connectivity within sensory and motor resting-state networks remains relatively stable in advanced age. Together, these patterns of age-related alterations in connectivity support the “last in, first out” hypothesis which suggests that brain regions that are the last to develop are the first to be affected by the aging process (Raz, 2000). In addition to CON, we also expected to find a relationship with DMN and FPCN connectivity and cognitive performance in our sample of healthy older adults.

In the present study, functional connectivity within DMN, FPCN, and DAN were not significantly related to the Fluid Cognition Composite. These results are surprising given the historic focus on the DMN as an individual network and its interactions with DAN and FPCN (Fox et al., 2005; Buckner et al., 2008; Ferreira and Busatto, 2013; Spreng et al., 2013). The most common finding in the resting-state and aging literature is that there are disruptions in resting-state functional connectivity within the DMN present in nonpathological aging and Alzheimer’s disease (Greicius et al., 2004; Buckner et al., 2008; Ferreira and Busatto, 2013; Ferreira et al., 2016). However, relating resting-state characteristics of the DMN and other networks to actual cognitive performance in older adults has resulted in mixed findings (Andrews-Hanna et al., 2007; Damoiseaux et al., 2007; Geerligs et al., 2015a; Ferreira et al., 2016). While disruptions in DMN resting-state connectivity may be a marker for memory deficits and Alzheimer’s disease pathology (Buckner, 2004; Buckner et al., 2008), our findings suggest that CON resting-state connectivity is a more robust marker for declines in attention-demanding cognitive domains present in nonpathological cognitive aging. This distinction between the roles of DMN and CON in the cognitive aging process may help facilitate better differentiation of nonpathological aging from disease states.

### Limitations and Future Directions

The present study is not without limitations regarding cohort characteristics and methodology. First, the age range of our older adult sample is relatively restricted (65–87). Therefore, we may only be representing connectivity and cognition relationships for a specific subset of older adults. Additionally, while our sample of older adults had an education range of 12–21 years, 67.5% of the sample obtained a bachelor’s degree or higher. Future work should examine this research question with a wider age range and a higher representation of older adults with education below 16 years to broaden the generalizability of these results. We conducted an ROI-ROI analysis to mitigate multiple comparisons and to limit our analyses to regions involved in established resting-state networks. Future studies should also examine voxel-wise approaches and whole-brain metrics to further explore the relationships found in the current study. Finally, we associated resting-state functional connectivity and cognitive performance in healthy older adults at one point in time. Therefore, we cannot make any conclusions about within-participant changes in connectivity and cognitive performance over time. In the course of neurodegenerative diseases, disrupted connectivity is apparent long before the presence of cognitive decline (Chen et al., 2016). As such, our findings may offer specific networks central to the early stages of cognitive aging. Future work should longitudinally assess changes in CON connectivity to see if disruptions in these networks are associated with the progression to MCI and Alzheimer’s disease.

## Conclusion

This study provides important new insights on inter-individual differences in resting-state network connectivity and the cognitive aging process. Notably, we provide evidence to suggest CON coherence as a potential new marker for fluid cognitive performance capacity in nonpathological aging. Out of the four higher-order cognitive networks, connectivity within the CON network exhibited the strongest relationship with a sensitive measure of general fluid cognitive ability in our large sample of healthy older adults. Furthermore, CON connectivity explained a greater percentage of the variance in fluid cognitive performance than both age and education. CON connectivity mapped onto three out of the five fluid cognitive subtests, reflecting a global rather than domain-specific influence on the cognitive aging process. Collectively, these results suggest that CON connectivity may be a central facet of the cognitive aging process and deserves increased focus in future research investigating the neural substrates of age-related cognitive decline.

## Data Availability Statement

Data are managed under the data-sharing agreement established with NIA and the parent R01 clinical trial Data Safety and Monitoring Board in the context of an ongoing Phase III clinical trial (ACT study, R01AG054077). All trial data will be made publicly available 2 years after completion of the parent clinical trial, per NIA and DSMB agreement. Requests for baseline data can be submitted to the ACT Publication and Presentation (P&P) Committee and will require submission of data use, authorship, and analytic plan for review by the P&P committee (ajwoods@phhp.ufl.edu).

## Ethics Statement

The studies involving human participants were reviewed and approved by Institutional Review Boards at the University of Florida and the University of Arizona. The patients/participants provided their written informed consent to participate in this study.

## Author Contributions

HH and AW contributed to the conception and design of the study. HH extracted the data, performed the statistical analyses, and wrote the first draft of the manuscript. AW, AO’S, JK, EB, and NE helped organize data and provide resources. EV, PB, and SS collected data. EP, GH, SW, SD, GA, MM, RC, and AW were involved in project administration. All authors contributed to manuscript revision, read, and approved the submitted version.

## Conflict of Interest

The authors declare that the research was conducted in the absence of any commercial or financial relationships that could be construed as a potential conflict of interest.

## Footnotes

^1^www.nihtoolbox.org

^2^www.nitrc.org/projects/artifact_detect
